# Effects of Fermented Compound Chinese Herbal Feed on Gut Microbiota, Immune Response, and Disease Resistance in Chinese Soft-Shelled Turtle (*Pelodiscus sinensis*)

**DOI:** 10.3390/ani16071054

**Published:** 2026-03-31

**Authors:** Chenxi Lu, Kangtao Cai, Xihua Chen, Zhen Wang, Huayou Chen, Ping Wu, Zhongjian Guo, Yong Feng

**Affiliations:** 1 School of the Life Sciences, Jiangsu University, 301 Xuefu Road, Zhenjiang 212013, China; 2 Key Laboratory of Fermentation Engineering (Ministry of Education), Hubei University of Technology, Wuhan 430068, China; 3 School of Medicine, Jiangsu University, 301 Xuefu Road, Zhenjiang 212013, China

**Keywords:** Chinese soft-shelled turtles, fermentation, Chinese herbs, antibacterial activity, *Pelodiscus sinensis*

## Abstract

In this study, Chinese medicinal herbs were evaluated as potential antibiotic substitutes for Chinese soft-shelled turtle (*Pelodiscus sinensis*). Forty-five herbs were initially screened for antibacterial activity against *Salmonella enteritidis*, *Escherichia coli*, and *Shigella flexneri*. Nine herbs exhibiting broad-spectrum inhibitory effects were selected and subjected to microbial fermentation, after which their antibacterial activities were reassessed and applied as dietary supplements in feeding trials. The results showed that fermentation altered the antibacterial activities of several herbs and enhanced their overall functional performance. Dietary supplementation with fermented Chinese herbal medicine did not adversely affect feed utilization but significantly improved hematological parameters, liver and kidney function indicators, antioxidant capacity, and nonspecific immune responses. Furthermore, turtles fed fermented herbal diets exhibited higher survival rates following bacterial challenge. Intestinal microbiota analysis based on 16S rRNA gene sequencing indicated that fermented herbal supplementation modulated microbial community structure by reducing potential pathogens and increasing beneficial bacterial taxa associated with intestinal health. These findings suggest that microbial fermentation effectively enhances the biological efficacy of Chinese medicinal herbs. Fermented herbal feed additives represent a promising green alternative to antibiotics for soft-shelled turtle aquaculture. The global ban on prophylactic antibiotics drives the need for safe, effective feed alternatives. Microbial fermentation of Chinese herbs (FCM) is proposed to enhance efficacy and detoxification, but its comprehensive effects in aquaculture require deeper investigation. This study evaluated compound unfermented (CM) and fermented (FCM) Chinese herbal supplements on the Chinese soft-shelled turtle (*Pelodiscus sinensis*). Initial screening showed fermentation generally enhanced the antibacterial activity of the herbs against common enteric pathogens (*S. enteritidis*, *E. coli*, *S. flexneri*). Results indicated that the FCM diet significantly improved physiological status, leading to higher red blood cell counts, better liver/kidney function (reduced ALT/AST, UREA), and stronger immune/antioxidant responses (increased Lysozyme and T-AOC) compared to CM or control diets. Critically, the FCM group achieved the highest survival rates across all single and combined pathogen challenges, demonstrating superior protective efficacy. Furthermore, FCM effectively modulated the gut microbiota, enriching beneficial fermentative bacteria. In conclusion, microbial fermentation significantly amplifies the health-promoting and protective benefits of Chinese herbal supplements in soft-shelled turtles, positioning FCM as a promising green alternative for disease control in aquaculture.

## Simple Summary

This study first screened 45 Chinese medicinal herbs for antibacterial activity against three common aquatic pathogens (*Salmonella enteritidis*, *Escherichia coli*, *Shigella flexneri*), selected nine herbs with broad-spectrum inhibition for microbial fermentation, and then conducted feeding trials to assess the effects of fermented and unfermented herbal supplements on Chinese soft-shelled turtle (*Pelodiscus sinensis*). Results showed that fermentation significantly enhanced the herbs’ antibacterial activity, and fermented herbal feed had no adverse effects on feed utilization. Compared with the control and unfermented groups, turtles fed fermented herbal diet had improved hematological indicators, liver and kidney function, antioxidant capacity and non-specific immunity, as well as higher survival rates after pathogen challenge; this supplement also optimized intestinal microbiota by reducing harmful bacteria and enriching beneficial strains. Overall, fermented Chinese herbal feed is a promising antibiotic alternative for healthy and sustainable soft-shelled turtle aquaculture.

## 1. Introduction

*Salmonella enteritidis*, *Escherichia coli*, and *Shigella flexneri* are among the most common zoonotic intestinal pathogens encountered in aquaculture systems [1,2,3]. Although these bacteria are often considered opportunistic, outbreaks can occur rapidly when environmental or husbandry conditions deteriorate, leading to severe enteric disease and substantial economic losses in cultured species [4,5]. In traditional farming practices, antibiotics have frequently been applied as a primary means of disease prevention and control. However, the long-term and sometimes indiscriminate use of antibiotics has raised serious concerns regarding antimicrobial resistance, drug residues, and ecological risks, prompting regulatory restrictions and an urgent need for effective antibiotic alternatives [6,7].

Chinese herbal medicines have long been used in animal production because of their broad antibacterial activity, relatively low toxicity, and additional benefits for immune regulation and growth performance [8,9]. Numerous studies have shown that herbal supplements can enhance host resistance to disease and improve physiological status in human, livestock and aquatic animals [10,11,12]. More recently, microbial fermentation has emerged as an effective strategy to further enhance the functionality of Chinese herbal medicines. Fermentation can degrade complex plant cell wall components such as cellulose, hemicellulose, lignin, and pectin through microbial extracellular enzymes, thereby facilitating the release and transformation of bioactive compounds [13,14,15,16,17]. At the same time, fermentation may reduce toxic or irritant constituents and generate new functional substances, including organic acids, antimicrobial peptides, and probiotic microorganisms capable of colonizing the intestinal tract [18,19,20,21,22,23].

Despite these advantages, research on fermented Chinese herbal medicines remains at an early stage [24,25]. Several key limitations persist, including an incomplete understanding of the transformation pathways of herbal constituents during fermentation, insufficient quantitative evaluation of toxin degradation, and a lack of systematic screening and justification for the selection of fermentation strains [26]. In many cases, fermentation processes are optimized empirically, without clear mechanistic explanations for efficacy enhancement or standardized quality control criteria [27,28]. Moreover, most existing studies focus on changes in one or a few known active components, while newly generated or less-characterized metabolites are often overlooked [29,30].

In animal production systems, fermented Chinese herbal feeds have shown encouraging results in livestock species such as pigs, poultry, and ruminants, as well as in several aquatic species. These studies have reported improvements in growth performance, reduced disease incidence, and modulation of intestinal microbiota [31,32]. However, studies focusing on Chinese soft-shelled turtles (*Pelodiscus sinensis*) remain limited. Therefore, the objective of the present study was to systematically screen Chinese medicinal herbs with antibacterial activity against common intestinal pathogens, develop a compound Chinese herbal feed based on selected candidates, and evaluate the effects of fermented and unfermented formulations on growth performance, immune function, physiological status, pathogen resistance, and gut microbiota in *P. sinensis*. This work aims to provide experimental evidence and mechanistic insight supporting the application of fermented Chinese herbal feed as a green and sustainable alternative to antibiotics in turtle aquaculture.

## 2. Materials and Methods

### 2.1. Materials and Microorganisms

In total, 45 kinds of Chinese herbs were purchased from Anhui Huzhou Chinese herbal market (Aspartame, Dogwood, Garlic, *Schisandra chinensis*, Coptis, Corydalis herb, Forsythia, *Polygonum cuspidatum*, Purslane, Catechu, Green bark, Anemarrhena, Chinensis, *Prunus chinensis*, Sapwood, Pulsatilla, Myrobalan, Wormwood leaf, Pomegranate peel, Black plum, Scutellaria, Astragalus, Phellodendron, *Herba japonica*, Gallnut, Wild chrysanthemum, Ligustrum, White atractylodes, *Radix isatidis*, Ginkgo leaf, Dandelion, Schizonepeta, *Angelica sinensis*, *Eucommia ulmoides*, Rhubarb, Red peony root, *Eucommia ulmoides* leaf, hawthorn, Rehmannia, Licorice, Sichuan dome, honeysuckle, Green leaf, Cocklebur seed, andrographitis, Soybean meal and starch). *Lactobacillus plantarum* (CGMCC 1.557) and *Lactobacillus rhamnosus* (CGMCC 1.2467) were purchased from the China General Microbiology Culture Collection. *Bacillus subtilis* and *Saccharomyces cerevisiae* were preserved by laboratory screening [13,33,34].

### 2.2. Preparation of Fermented Chinese Herbal Additive

The fermentation medium was herbal medicine (one of 45 species, 50 g), soybean meal (30 g), and secondary flour (20 g), and a solution of the above five fermenting bacteria (*Bacillus subtilis*, *Saccharomyces cerevisiae*, *Lactobacillus plantarum*, *Lactobacillus rhamnosus*, and *Lactobacillus fermentum*) was inoculated into the fermentation medium at an inoculum level of 5% (*v*/*w*). The colony forming units in the fermentation broth were approximately 10^8^ /mL (CFU/mL). The medium moisture was adjusted to 45% (*v*/*w*), and the medium was sealed in a fermentation bag with a one-way exhaust valve and fermented anaerobically at 30 °C for 7 days.

### 2.3. Antimicrobial Activity Evaluation In Vitro to Fermented Chinese Herbs Additive

Solid media plate inhibition circle method to determine the degree of inhibition of each herbal medicine (for use as defined by the Clinical and Laboratory Standards Institute (CLSI)) [35,36]. Take 1 g of each plant fermented feed, put it into a 50 mL conical flask, add 10 mL of 60% methanol solution and extract for 30 min, filter and take the supernatant as feed extract. A total of 200 µL of pathogenic bacteria solution (10^8^ CFU/mL) was applied to the surface of nutrient agar plates. After drying, a 6 mm filter paper disc was attached and 10 µL of feed extract was added dropwise to the filter paper for absorption. 60% methanol was used as a negative control and 0.2 mg/mL kanamycin was used as a positive antibiotic control. The plates were diffused in a refrigerator at 4 °C for 4 h and removed and incubated in an incubator at 37 °C for 12 h. After the incubation was completed, the size of the inhibition circle was observed and the diameter of the inhibition circle was measured.

### 2.4. Feed Preparation and Breeding Design

Local healthy and active Chinese soft-shelled turtles with an average body weight of 400 ± 20 g were selected for the feeding trial. A total of 60 turtles were randomly assigned to three dietary treatments: a fermented compound Chinese herbal diet (FCM), an unfermented compound Chinese herbal diet (CM), and a control diet (CG) without herbal supplementation. Each treatment consisted of three independent replicates (rearing units), and turtles were randomly distributed to ensure true experimental replication. Each replicate tank measured 1.8 m × 9 m × 0.6 m, with a stocking density of 1.0–1.2 individuals per square meter.

The detailed feed formulation is provided in Table S1. Soft pellets were fed twice daily (11:00–14:00 and 19:00–22:00) at 2% of body weight. Uneaten feed was removed by siphoning 1 h after feeding to ensure accurate feed intake calculation. During the 60-day experimental period, water quality parameters were maintained as follows: pH 7.0–8.5, dissolved oxygen 3–5 mg/L, ammonia nitrogen < 2 mg/L, and water temperature maintained at 30 °C using a thermostatic heating rod. The photoperiod was controlled at 12 h light per day, with light intensity maintained at 3000–5000 lx.

### 2.5. Production Performance of Chinese Soft-Shelled Turtles

The total weight of each Chinese soft-shelled turtle was recorded at the beginning and end of the experiment. The feed consumption of each group was recorded daily. At the end of the experiment, Chinese soft-shelled turtles were sampled after fasting for 24 h to empty their intestines. The following variables were calculated:

Weight gain rate (WG, g) = (final weight − initial weight)/initial weight

Feed conversion rate (FCR, %) = dry feed weight/(final weight − initial weight)

### 2.6. Blood Hematological Parameters of Chinese Soft-Shelled Turtle

Before feeding on Day 61, blood samples were collected from the jugular vein of 10 soft-shelled turtles in each group. Approximately 5 mL of blood from each turtle was used for routine hematological analysis. An additional 5 mL of blood was collected, allowed to clot at room temperature for 30 min, and centrifuged at 3000 rpm for 10 min to obtain serum. Red blood cells (RBC) and white blood cells (WBC) were counted using a hemocytometer following standard procedures [37]. Serum biochemical parameters-including alanine aminotransferase (ALT), aspartate aminotransferase (AST), urea, uric acid (UA), glucose (GLU), total cholesterol (TCHOL), high-density lipoprotein cholesterol (HDL-C), and low-density lipoprotein cholesterol (LDL-C)-were measured using a fully automated biochemical analyzer (Model: Beckman Coulter AU480 Manufacturer: Beckman Coulter Experimental Systems (Suzhou) Co., Ltd., Suzhou, China) with commercially available diagnostic reagent kits, following the manufacturer’s instructions [38,39].

### 2.7. Determination of Serum Antioxidant Immune Indexes of Chinese Soft-Shelled Turtle

The content of lysozyme, total antioxidant capacity (T-AOC), acid phosphatase (ACP) and alkaline phosphatase (AKP) in serum was determined by referring to the experimental methods in the procurement manual of [40], and the serum of Chinese soft-shelled turtle was collected as above.

### 2.8. Challenge Experiment of S. enteritidis, E. coli and S. flexneri

This experiment used local healthy Chinese soft-shelled turtle juveniles with an average weight of 20 ± 5 g. The test was divided into two groups, with the test group adding 90% Basal diet on top of 10% compound fermented Chinese herbs and the control group adding only normal feed.

Prior to the test, each group was fed a conventional diet for 1 week. The feed was then supplemented with each enteropathogenic bacterium at an average feeding rate of 10^6^ CFU per turtle. During the trial period, the feed was fed daily from 11:00–14:00 and 19:00–22:00 at 2% of the body mass of the turtle. After 1 h of feeding, the uneaten feed was cleaned by siphoning.

### 2.9. Statistical Analysis

Statistical analysis of all data for each parameter was performed using SPSS version 11.5 (SPSS 26.0, Chicago, IL, USA) followed by a one-way ANOVA with a significance threshold of (*p* < 0.05). The above experiment was repeated five times (the test for determining the inhibition zone of fermented traditional Chinese medicine was conducted independently five times), and the results are expressed as mean ± standard deviation.

### 2.10. Animal Ethics and Euthanasia Standards

All experimental procedures were conducted in accordance with the general principles of the Guidelines for the Care and Use of Laboratory Animals (NIH), particularly the 3R principles (replacement, reduction, refinement), and complied with the Animal Ethics Review Standards of the Institutional Animal Care and Use Committee of Jiangsu University (UJS IACUC). The experimental protocol was reviewed and approved by the Animal Ethics Committee of Jiangsu University (Approval No.: UJS-IACUC-2021081401).

### 2.11. Anesthesia and Euthanasia Procedures

Soft-shelled turtles were anesthetized via intraperitoneal injection of sodium pentobarbital (80–100 mg/kg body weight). Adequate anesthesia was confirmed by the absence of corneal reflex, limb withdrawal reflex, and neck retraction reflex. After complete anesthesia was verified, cervical dislocation was performed rapidly and gently to ensure a humane death. Death was confirmed by the cessation of respiration, heartbeat, and cloacal pulse. Intestinal tissues for 16S microbiome analysis were collected immediately after confirmation of death to preserve sample integrity.

## 3. Results and Discussion

### 3.1. Screening of Antibacterial Activities of Chinese Herbal Materials Against Intestinal Pathogens

To identify suitable herbal components for the development of a compound Chinese herbal feed with antibacterial activity, the inhibitory effects of individual Chinese medicinal materials against three common intestinal pathogens were evaluated. Against *Salmonella enteritidis*, 19 out of 45 tested Chinese herbal materials exhibited measurable inhibitory activity prior to fermentation (Table 1). Among these, Coptis, *Schisandra chinensis*, *Anemarrhena asphodeloides*, and gallnut showed relatively strong inhibitory effects. Following fermentation, antibacterial activity was detected in most herbal materials, with the exception of black plum. Compared with the unfermented state, the inhibition zones of most herbs were further enhanced after fermentation, indicating that microbial fermentation generally improved antibacterial efficacy against *S. enteritidis*.

In assays against *Escherichia coli*, only three herbs (Myrobalan, gallnut, and *Andrographis paniculata*) exhibited detectable inhibitory activity before fermentation (Table 2). After fermentation, the number of herbs showing antibacterial effects increased markedly to 18. In most cases, fermentation resulted in enlarged inhibition zones compared with the unfermented counterparts, with particularly notable enhancement observed for gallnut, green bark, *Fructus chinensis*, and licorice. For *Shigella flexneri*, antibacterial activity before fermentation was observed in only four herbs (catechu, myrobalan, gallnut, and wild chrysanthemum) (Table 3). After fermentation, the number of herbs exhibiting inhibitory effects increased to eight, and the majority showed enhanced inhibition zone diameters relative to the unfermented state. These results suggest that microbial fermentation expanded both the spectrum and intensity of antibacterial activity of several Chinese herbal materials against *S. flexneri*.

The observed enhancement or maintenance of antibacterial activity following fermentation may be attributed to multiple factors. Microbial fermentation can modify or increase the availability of active herbal constituents, thereby influencing inhibitory efficacy. In addition, organic acids produced during probiotic fermentation [41], as well as fermentation-derived antimicrobial peptides [42], may further contribute to the suppression of pathogenic bacterial growth.

Previous studies have reported that many of the selected herbs are rich in bioactive compounds such as baicalin, baicalein, tannins, and gallic acid, all of which exhibit broad-spectrum antibacterial properties [43]. Consistent with this, Wang and colleagues reported that baicalin in *Scutellaria baicalensis* can be converted into baicalein through fermentation by β-glucosidase–producing strains [44]. Similarly, tannase generated during microbial fermentation can hydrolyze tannins to yield gallic acid [45], which may further enhance antibacterial efficacy. Collectively, these transformations may underlie the sustained or improved inhibitory effects observed for several fermented herbal materials.

Based on their collective inhibitory effects against all three tested pathogens, nine herbal materials—*Coptis chinensis*, *Schisandra chinensis*, *Anemarrhena*, gallnut, Rhubarb, Scutellaria, licorice, Myrobalan, and Catechu—were selected for subsequent fermentation. Although fermentation led to a reduction in inhibition zone diameter for certain herbs to certain pathogens (e.g., gallnut to *S. enteritidis*, and *Terminalia chebula* to *S. flexneri*), These materials retained relatively strong antibacterial activity after fermentation, with inhibition zones remaining larger than those of most other herbs.

### 3.2. Effects of Fermented and Unfermented Chinese Herbal Diets on Production Performance

To evaluate the efficacy of incorporating herbal supplements into commercial diets, we assessed how the substitution of standard feed with fermented or unfermented Chinese herbal medicine impacted the feed efficiency of *P. sinensis*. The feed efficiency recorded for *P. sinensis* under different dietary substitutions are detailed in Table 4. The results indicate that replacing 10% of the commercial feed with either fermented or unfermented Chinese herbal medicine resulted in varied outcomes. Specifically, no significant difference in feed efficiency was observed between the fermented group and the full-price powder (control) group. However, the unfermented group exhibited a significantly higher feed efficiency compared to the other two groups.

This higher feed efficiency in the unfermented group is likely attributable to the predominance of lignocellulosic components, which are generally difficult for the turtle’s digestive system to absorb and utilize without prior enzymatic breakdown [46,47]. Bacterial enzymes, present in the fermentation process, are essential for degrading lignocellulose into digestible sugars and breaking down crude protein into smaller peptides [16,48]. Furthermore, the inclusion of fermented herbs may introduce beneficial enzymes that aid in the degradation and transformation of nutrients within the primary diet [49]. It is also plausible that probiotic strains present in the fermented Chinese herbal medicine supplement colonize the turtle’s intestine upon ingestion, thereby enhancing overall intestinal digestion and absorption capacity [50]. This colonization not only influences the structural environment of the gut but also favorably modifies the composition of the intestinal microflora, collectively leading to improved nutritional assimilation [51].

### 3.3. Effects of Fermented and Unfermented Chinese Herbal Diets on the Number of RBC and WBC

**Figure 1:**
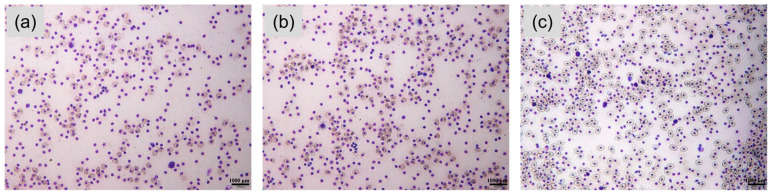
Blood cell morphology of Chinese soft-shelled turtles. (**a**) control group, (**b**,**c**) Chinese herbs feed group before (**b**) and after fermentation (**c**).

To assess the effects of compound Chinese herbal supplementation, with or without fermentation, on hematological parameters in Chinese soft-shelled turtles, blood cell morphology and counts were analyzed. Blood smear microscopy revealed that leukocyte abundance was lower in both the Chinese herbal medicine group and the fermented compound herbal medicine group than in the full-price fish meal control group (Figure 1, Table 5). Leukocytes play essential roles in immune responses, and elevated leukocyte levels are often associated with bacterial infection or inflammatory stress [52]. The reduced leukocyte counts observed in the herbal-supplemented groups may therefore indicate that bioactive components in the compound herbs alleviated inflammatory status and, to some extent, suppressed bacterial infection in *P. sinensis*. However, it should be noted that leukopenia may also reflect immunosuppression, altered leukocyte mobilization, or physiological stress responses.

In contrast, erythrocyte counts showed an opposite trend. The erythrocyte number in the unfermented herbal medicine group was moderately higher than that in the control group, whereas a significantly higher erythrocyte count was observed in the fermented compound herbal medicine group compared with both other groups (Figure 1, Table 5). This enhancement may be related to microbial-fermentation-derived changes in the herbal feed. Previous studies have demonstrated that probiotic fermentation can promote the formation of ion-chelating peptides, which effectively protect divalent iron from oxidation during fermentation and improve its bioavailability [13]. Improved iron absorption can, in turn, facilitate hemoglobin synthesis and erythropoiesis. Accordingly, the elevated erythrocyte levels observed in the fermented herbal group suggest that fermentation of compound Chinese herbs may enhance iron utilization and red blood cell production in Chinese soft-shelled turtles.

### 3.4. Effects of Fermented and Unfermented Chinese Herbal Diets on Serum Biochemical Parameters

To evaluate whether dietary supplementation with Chinese herbs, particularly after microbial fermentation, affects systemic metabolic status and organ function in *P. sinensis*, serum biochemical parameters related to liver function, renal function, and lipid metabolism were analyzed (Table 6). Activities of alanine aminotransferase (ALT) and aspartate aminotransferase (AST), two commonly used indicators of hepatic function, were highest in the Basal diet group, intermediate in the Chinese medicine (CM) group, and lowest in the fermented Chinese medicine (FCM) group. This gradient suggests that dietary supplementation with Chinese herbs exerted a protective effect on liver function, which was further enhanced after microbial fermentation. Similar hepatoprotective effects have been reported previously, Zhang et al. demonstrated that baicalein supplementation alleviated infection-induced transaminase elevation in piglets [53]. An additional explanation may be related to the high abundance of viable probiotics in the fermented herbal feed (up to 10^9^ CFU g^−1^). Previous studies have shown that dietary supplementation with compound probiotics, including *B. subtilis* and *L. casei*, significantly reduced serum transaminase activities in Chinese soft-shelled turtles, possibly through intestinal colonization and the production of beneficial secondary metabolites [54,55].

Serum urea (UREA), uric acid (UA), and creatinine (CREA), which are closely associated with renal function [56], showed different patterns among groups. UREA and UA concentrations were lowest in the FCM group, while UREA levels in the CM group were lower than those in the control group; no significant difference in UA was observed between the CM and control groups. These results indicate that Chinese herbal supplementation contributed to improved renal metabolic status, with fermentation further strengthening this effect. Similar findings have been reported in other animals, where herbal formulations containing *S. baicalensis* exhibited diuretic properties and alleviated hyperuricemia, effects that may be enhanced after fermentation.

In terms of lipid metabolism, both high-density lipoprotein cholesterol (HDL-C) and low-density lipoprotein cholesterol (LDL-C) were higher in the FCM group than in the other two groups. The elevated HDL-C level suggests that fermented herbal supplementation may positively modulate cholesterol metabolism in *P. sinensis*. This effect may be associated with probiotic-mediated regulation of intestinal microbiota, which has been shown to influence systemic lipid and glucose metabolism through microbial secondary metabolites [57]. In addition, certain bioactive components of Chinese herbs may directly contribute to lipid regulation. For instance, flavonoids derived from *Chrysanthemum* have been reported to reduce serum TC, TG, and LDL-C while increasing HDL-C, accompanied by enhanced antioxidant capacity [58].

Overall, the serum biochemical profiles indicate that compound Chinese herbal supplementation, particularly after fermentation, exerted beneficial effects on hepatic and renal function as well as lipid metabolism in Chinese soft-shelled turtles, without evidence of adverse physiological disturbance.

### 3.5. Effects of Fermented and Unfermented Chinese Herbal Diets on Serum Immune and Antioxidant Indices

Given the importance of immune defense and antioxidant capacity for disease resistance in Chinese soft-shelled turtles, the effects of dietary supplementation with fermented and unfermented compound Chinese herbs on serum immune and antioxidant indices were assessed. Lysozyme activity (LSM), an indicator of non-specific antibacterial defense [59], was higher in the fermented Chinese medicine group (FCM; 1033.22 ± 45.16 U·mL^−1^) and the unfermented Chinese medicine group (CM; 817.32 ± 40.16 U·mL^−1^) than in the control group (CG; 533.10 ± 32.11 U·mL^−1^), corresponding to approximately 1.93- and 1.53-fold increases, respectively (Table 7). Total antioxidant capacity (T-AOC) showed a similar pattern, with higher values in the FCM (3.54 ± 0.12 U·mL^−1^) and CM (3.29 ± 0.32 U·mL^−1^) groups compared with the control (2.30 ± 0.14 U·mL^−1^). Together, these results indicate that both Chinese herbal supplementation and microbial fermentation enhanced systemic antioxidant status and basal non-specific immune markers in Chinese soft-shelled turtles, with the fermented preparation producing the greatest overall responses.

Chinese soft-shelled turtles are rich in polyunsaturated fatty acids, which are susceptible to oxidative damage and may predispose the organism to disease. Previous studies have shown that antioxidant capacity is closely associated with non-specific immune function, and that enhancement of antioxidant defenses can stimulate immune responses and improve disease resistance [60]. Accordingly, serum T-AOC provides an indirect indicator of immune status in this species. In the present study, T-AOC values in the fermented Chinese medicine and Chinese medicine groups were approximately 1.53-fold and 1.43-fold higher than those of the control group, respectively, suggesting that compound Chinese herbal supplementation effectively enhanced antioxidant capacity, with a stronger effect observed after fermentation. Similar findings have been reported in studies of probiotic anaerobically fermented feeds, which were shown to protect divalent iron ions from oxidation and enhance antioxidant capacity following intestinal absorption [13,61].

Acid phosphatase (ACP), a lysosomal marker enzyme in macrophages that has been linked to tissue damage mechanisms [62], did not differ notably among groups (FCM: 1.84 ± 0.18 U·mL^−1^; CM: 1.84 ± 0.05 U·mL^−1^; CG: 1.88 ± 0.15 U·mL^−1^). This suggests that dietary supplementation with either fermented or unfermented Chinese herbal feeds did not induce detectable lysosomal damage under the experimental conditions. Alkaline phosphatase (AKP), an enzyme involved in dorsal carapace formation as well as calcium and phosphorus metabolism in Chinese soft-shelled turtles [63], exhibited the highest activity in the control group (5.55 ± 0.23 U·mL^−1^), with lower values observed in the FCM (4.49 ± 0.11 U·mL^−1^) and CM (4.33 ± 0.14 U·mL^−1^) groups. This pattern may reflect differences in dietary composition, such as mineral availability, or altered intestinal and hepatic metabolism following herbal supplementation and fermentation, rather than adverse physiological effects.

Overall, these results demonstrate that dietary supplementation with compound Chinese herbs enhanced both immune and antioxidant status in Chinese soft-shelled turtles. Increases in serum lysozyme activity and total antioxidant capacity indicate reinforcement of non-specific immune defense and systemic redox balance. Compared with unfermented herbal preparations, fermentation further modulated these responses, potentially by improving the bioavailability of functional components and generating fermentation-derived metabolites with antioxidant or immunomodulatory properties. The absence of marked changes in ACP activity suggests good physiological tolerance to long-term supplementation, while the moderate changes in AKP activity are more likely attributable to diet-related metabolic adjustments than to detrimental effects.

### 3.6. Effects of Fermented and Unfermented Chinese Medicinal Feeds on Survival Under Bacterial Challenge

To evaluate the effects of Chinese medicinal supplementation, with or without fermentation, on turtle survival under pathogenic challenge, turtles were exposed to *S. enteritidis*, *E. coli*, *S. fowleri*, and a combination of these three pathogens. In the single-pathogen challenge with *S. enteritidis* (Table 8), survival rates increased from 33.33% in the control group to 66.67% in the unfermented Chinese medicine group and further to 96.67% in the fermented Chinese medicine group. A similar trend was observed in the *E. coli* challenge (Table 9), where the fermented Chinese medicine group achieved a survival rate of 90.00%, compared with 83.33% in the unfermented Chinese medicine group and 50.00% in the control group. These results indicate that dietary supplementation with Chinese medicinal components substantially improved resistance to enteric bacterial infection, and that fermentation further enhanced this protective effect.

The protective advantage of fermented Chinese medicinal feed was particularly evident in the *S. fowleri* challenge (Table 10). Survival in the control group was limited to 20.00%, whereas turtles fed Chinese medicine and fermented Chinese medicine diets showed survival rates of 56.67% and 80.00%, respectively. This pronounced difference suggests that the fermented formulation was more effective in mitigating the pathogenic effects of *S. fowleri* than the unfermented herbal preparation. Under the combined challenge with *S. enteritidis*, *E. coli*, and *S. fowleri* (Table 11), overall survival decreased in all groups, reflecting the increased severity of mixed infection. Nevertheless, the fermented Chinese medicine group maintained the highest survival rate (63.33%), which was nearly double that of the Chinese medicine group (33.33%) and substantially higher than that of the control group (13.33%). These findings demonstrate that the fermented herbal feed provided more robust protection under conditions of complex pathogenic stress.

Collectively, the challenge experiments consistently showed that both Chinese medicinal supplementation and its fermented form improved survival of Chinese soft-shelled turtles exposed to intestinal pathogenic bacteria, with fermentation conferring an additional protective advantage. The superior performance of the fermented Chinese medicine feed across single and combined pathogen challenges suggests that fermentation enhances the functional efficacy of Chinese medicinal diets in improving disease resistance [64], particularly under high pathogen pressure.

### 3.7. Herbal Supplementation Reshapes Gut Microbial OTU Profiles

**Figure 2:**
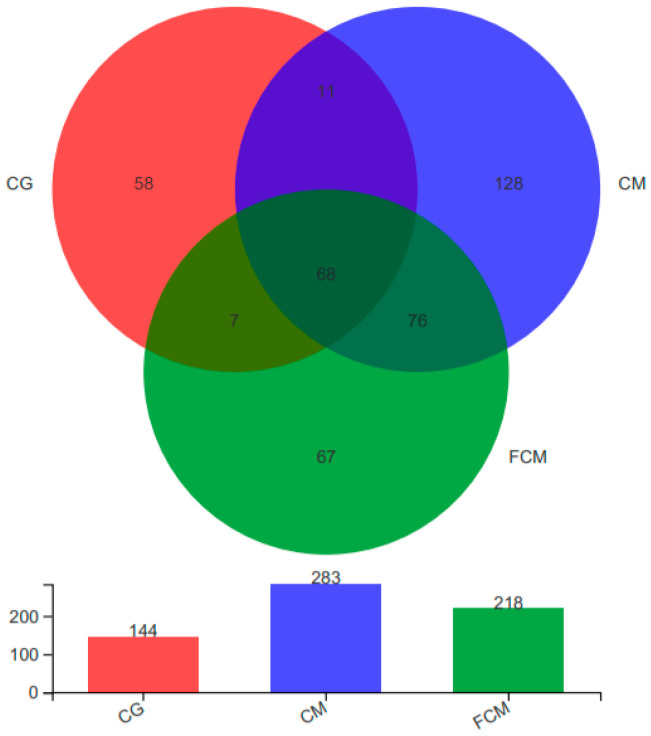
Venn diagram and total OTU counts of gut microbiota from three turtle groups. CG, control group; CM, Chinese medicine group; FCM, fermented Chinese medicine group.

To investigate the effects of Chinese medicinal supplementation, with or without fermentation, on the gut microbiota of turtles, samples from three experimental groups were subjected to high-throughput 16S rRNA gene sequencing. The bacterial sequences were processed to remove redundancy, followed by OTU clustering at 97% similarity. Species composition analysis indicated that a total of 68 OTUs were shared by all groups (Figure 2). Exclusive pairwise overlaps were 11 OTUs between CM and CG, 7 OTUs between CG and FCM, and 76 OTUs between CM and FCM (each value excludes the 68 OTUs common to all groups). Unique OTUs numbered 128 for CM, 67 for FCM and 58 for CG. Accordingly, total OTU richness per sample group was 283 (CM), 218 (FCM) and 144 (CG). These results indicate that both Chinese herbal supplementation and its fermented form increased gut microbial richness compared with the control, with the unfermented herb group showing the greatest OTU richness. The substantial overlap between CM and FCM suggests that fermentation preserved many herb-associated taxa while also introducing or enriching unique taxa in the fermented product.

### 3.8. Effects of Fermented and Unfermented Chinese Medicine on Gut Microbial Composition

**Figure 3:**
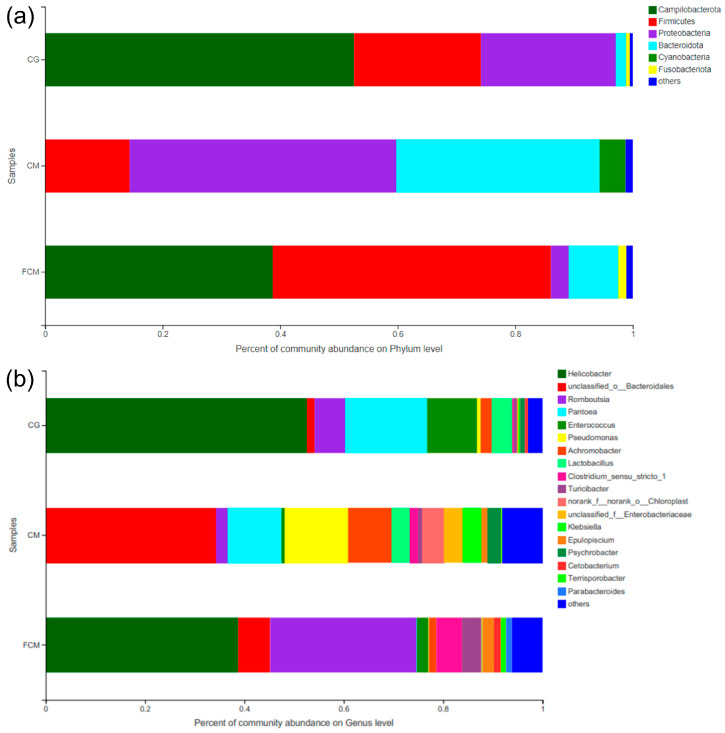
Bacterial community composition of turtle gut microbiota at the phylum (**a**) and genus (**b**) levels among the control group (CG), Chinese medicine group (CM), and fermented Chinese medicine group (FCM).

To investigate the effects of Chinese medicinal supplementation, with or without fermentation, on the gut microbiota community structure, the distribution patterns of bacterial communities were analyzed. At the phylum level (Figure 3a), *Campylobacterota* was one of the dominant bacterial phyla in the control group. Its relative abundance was markedly reduced in the Chinese medicine group and moderately reduced in the fermented Chinese medicine group. *Campylobacterota* includes several Gram-negative intestinal pathogens [65], and its decrease may reflect a suppressive effect of herbal supplementation on potentially harmful taxa. Notably, the reduction observed in the fermented group was less pronounced than that in the unfermented group, suggesting that fermentation may partially alter or reduce the activity of antibacterial components targeting this phylum.

The relative abundance of *Proteobacteria* was also lower in both Chinese medicine-treated groups compared with the control. *Proteobacteria* comprise numerous opportunistic pathogens, including *Vibrio* and *Helicobacter*, and their enrichment is commonly associated with intestinal dysbiosis and inflammation [66] A reduction in *Proteobacteria* may therefore indicate an improvement in gut microbial homeostasis following herbal supplementation. *Fusobacteriota* was detected mainly in the fermented Chinese medicine group, while it was absent or present at very low levels in the control and unfermented Chinese medicine groups. Although some members of *Fusobacteriota* are regarded as opportunistic pathogens [66], others have been reported to participate in carbohydrate fermentation and short-chain fatty acid production, which may confer benefits to the host [67].

The relative abundance of *Bacteroidota* increased in both herbal treatment groups, with a more pronounced increase in the unfermented Chinese medicine group, where it became one of the dominant phyla. In contrast, *Firmicutes* showed divergent responses: its proportion decreased in the Chinese medicine group but increased markedly in the fermented Chinese medicine group. These contrasting patterns indicate that fermentation altered the regulatory effects of Chinese medicinal supplementation on the *Firmicutes/Bacteroidota* balance, potentially reflecting differences in substrate availability and microbial metabolic pathways.

At the genus level (Figure 3b), distinct microbial profiles were observed among the three groups. The Chinese medicine group exhibited a higher number of detectable genera, indicating a more complex bacterial community structure compared with the control and fermented groups. The relative abundance of *Helicobacter* was high in the control group but was markedly reduced in the Chinese medicine group, while only a moderate decrease was observed in the fermented Chinese medicine group. Given that *Helicobacter* spp. are commonly regarded as intestinal pathogens, these results suggest that unfermented Chinese medicinal supplementation may exert a stronger inhibitory effect on this genus, whereas fermentation may partially attenuate this activity.

In contrast, *Romboutsia* and *Terrisporobacter* were enriched in the fermented Chinese medicine group but showed lower relative abundances in the unfermented group. These genera have been associated with fermentative metabolism and intestinal health in aquatic animals, suggesting that probiotic-mediated fermentation may promote the growth of functionally beneficial taxa. Several other genera, including *Parabacteroides*, *Turicibacter*, and *Clostridium sensu stricto 1*, showed increased relative abundances in both herbal treatment groups, particularly after fermentation. These taxa are commonly linked to bile acid transformation, short-chain fatty acid production, and fermentative metabolic processes, indicating potential benefits to host intestinal function [68].

Finally, *Pseudomonas*, a genus characterized by low virulence but high antibiotic resistance [69], was reduced in the fermented Chinese medicine group but increased in the unfermented Chinese medicine group. This pattern suggests that microbial fermentation may enhance the suppressive effect of Chinese medicinal supplementation on opportunistic bacteria, while unfermented herbal components alone may not be sufficient to inhibit this genus.

## 4. Conclusions

This study demonstrates that dietary supplementation with compound Chinese herbs markedly improves immune function, antioxidant capacity, and resistance to intestinal pathogens in Chinese soft-shelled turtles, and that microbial fermentation further enhances these beneficial effects. Compared with unfermented herbal feed, fermented Chinese herbal supplementation more effectively increased serum lysozyme activity and total antioxidant capacity, reshaped gut microbial communities by suppressing potential pathogens and promoting beneficial taxa, and significantly improved survival rates under bacterial challenge. Importantly, no evidence of physiological damage was observed, indicating good biosafety during long-term feeding. The enhanced efficacy of fermented herbs is likely attributable to improved bioavailability of active compounds and the generation of fermentation-derived metabolites with immunomodulatory and antimicrobial properties. Overall, these findings provide experimental support for the application of fermented compound Chinese herbal feed as a natural and effective alternative to antibiotics, offering a feasible strategy for improving health management and promoting sustainable development in turtle aquaculture.

## Figures and Tables

**Table 1:** Size of inhibition zone of Chinese herbal to *S. enteritidis* before and after fermentation.

Types of Fermented Herbs	Diameter of Inhibition Circle Before Fermentation (mm)	Diameter of Inhibition Circle After Fermentation (mm)	Types of Fermented Herbs	Diameter of Inhibition Circle Before Fermentation (mm)	Diameter of Inhibition Circle After Fermentation (mm)
Aspartame	7.00 ± 0.10 ^A^	10.0 ± 0.00 ^B^*	*Herba japonica*	/	9.00 ± 0.50 ^A^*
Dogwood	7.50 ± 0.50 ^A^	11.0 ± 0.50 ^B^*	Gallnut	14.00 ± 0.50 ^B^	8.60 ± 1.40 ^A^*
Garlic	8.25 ± 1.25 ^A^	9.00 ± 0.00 ^A^*	Wild chrysanthemum	/	7.50 ± 0.50 ^A^*
*Schisandra chinensis*	11.05 ± 0.05 ^B^	9.00 ± 0.15 ^A^*	Ligustrum	/	8.00 ± 0.10 ^A^*
Coptis	18.00 ± 0.05 ^B^	18.00 ± 0.05 ^C^	White atractylodes	/	9.00 ± 0.50 ^A^*
Corydalis herb	8.00 ± 0.05 ^A^	7.00 ± 0.30 ^A^*	*Radix isatidis*	/	8.50 ± 0.50 ^A^*
Forsythia	8.50 ± 0.00 ^A^	7.00 ± 0.00 ^A^*	Ginkgo leaf	/	9.00 ± 0.50 ^A^*
*Polygonum cuspidatum*	7.25 ± 1.25 ^A^	8.50 ± 0.25 ^A^*	Dandelion	/	9.00 ± 0.50 ^A^*
Purslane	8.00 ± 0.05 ^A^	8.00 ± 0.15 ^A^	Schizonepeta	/	7.75 ± 0.25 ^A^*
Catechu	8.00 ± 0.00 ^A^	8.90 ± 0.10 ^A^*	*Angelica sinensis*	/	6.25 ± 0.05 ^A^*
Green bark	7.20 ± 0.20 ^A^	8.50 ± 0.50 ^A^*	*Eucommia ulmoides*	/	6.90 ± 0.10 ^A^*
Anemarrhena	11.00 ± 0.10 ^B^	8.50 ± 0.50 ^A^*	Rhubarb	/	7.15 ± 0.35 ^A^*
Chinensis	7.00 ± 0.20 ^A^	8.00 ± 0.15 ^A^*	Red peony root	/	7.00 ± 0.00 ^A^*
*Prunus chinensis*	8.50 ± 0.00 ^A^	7.25 ± 0.25 ^A^*	*Eucommia ulmoides* leaf	/	6.60 ± 0.40 ^A^*
Sapwood	8.20 ± 0.10 ^A^	8.20 ± 0.10 ^A^	hawthorn	/	7.00 ± 0.10 ^A^*
Pulsatilla	7.00 ± 0.10 ^A^	8.00 ± 0.50 ^A^*	Rehmannia	/	6.50 ± 0.50 ^A^*
Myrobalan	/	7.20 ± 0.50 ^A^*	Licorice	8.00 ± 0.00 ^A^	9.00 ± 0.00 ^A^*
Wormwood leaf	/	8.00 ± 0.00 ^A^*	Sichuan dome	7.50 ± 0.50 ^A^	8.50 ± 0.50 ^A^*
Pomegranate peel	/	6.50 ± 0.00 ^A^*	honeysuckle	/	8.50 ± 0.50 ^A^*
Black plum	/	/	Green leaf	/	7.50 ± 0.50 ^A^*
Scutellaria	/	7.80 ± 0.50 ^A^*	Cocklebur seed	/	8.00 ± 0.00 ^A^*
Astragalus	/	7.00 ± 0.10 ^A^*	andrographitis	/	7.05 ± 0.05 ^A^*
Phellodendron	/	7.60 ± 0.20 ^A^*	Soybean meal and starch	/	8.00 ± 0.00 ^A^*

Note: Data are presented as mean ± SD. Within the same column, values sharing the same uppercase letter do not differ significantly (*p* > 0.05), whereas different uppercase letters indicate significant differences (*p* < 0.05). For paired comparisons within the same row (before vs. after fermentation), the absence of an asterisk (*) indicates no significant difference, while the presence of an asterisk (*) after fermentation indicates a significant difference (*p* < 0.05). A slash (/) indicates that no inhibition zone was detected.

**Table 2:** Size of inhibition zone of Chinese herbal medicine feed to *E. coli* before and after fermentation.

Types of Fermented Herbs	Diameter of Inhibition Circle Before Fermentation (mm)	Diameter of Inhibition Circle After Fermentation (mm)	Types of Fermented Herbs	Diameter of Inhibition Circle Before Fermentation (mm)	Diameter of Inhibition Circle After Fermentation (mm)
Aspartame	/	/	*Herba japonica*	/	/
Dogwood	/	/	Gallnut	8.00 ± 0.35 ^B^	9.00 ± 0.45 ^B^*
Garlic	/	/	Wild chrysanthemum	/	/
*Schisandra chinensis*	/	/	Ligustrum	/	/
Coptis	/	/	White atractylodes	/	/
Corydalis herb	/	/	*Radix isatidis*	/	/
Forsythia	/	/	Ginkgo leaf	/	/
*Polygonum cuspidatum*	/	/	Dandelion	/	/
Purslane	/	/	Schizonepeta	/	8.00 ± 0.15 ^A^*
Catechu	/	/	*Angelica sinensis*	/	8.00 ± 0.25 ^A^*
Green bark	/	9.00 ± 0.25 ^B^*	*Eucommia ulmoides*	/	8.00 ± 0.35 ^A^*
Anemarrhena	/	/	Rhubarb	/	9.00 ± 0.30 ^B^*
Chinensis	/	9.00 ± 0.15 ^B^*	Red peony root	/	7.00 ± 0.15 ^A^*
*Prunus chinensis*	/	9.00 ± 0.30 ^B^*	*Eucommia ulmoides* leaf	/	7.00 ± 0.20 ^A^*
Sapwood	/	8.15 ± 0.15 ^A^*	hawthorn	/	/
Pulsatilla	/	8.50 ± 0.25 ^A^*	Rehmannia	/	7.00 ± 0.25 ^A^*
Myrobalan	6.5 ± 0.15 ^A^	8.50 ± 0.45 ^A^*	Licorice	/	9.00 ± 0.35 ^B^*
Wormwood leaf	/	8.50 ± 0.10 ^A^*	Sichuan dome	/	/
Pomegranate peel	/	/	honeysuckle	/	/
Black plum	/	/	Green leaf	/	/
Scutellaria	/	9.00 ± 0.30 ^B^*	Cocklebur seed	/	/
Astragalus	/	/	Andrographitis	6.50 ± 0.20 ^A^	7.00 ± 0.15 ^A^*
Phellodendron	/	/	Soybean meal and starch	/	/

Note: Data are presented as mean ± SD. Within the same column, values sharing the same uppercase letter do not differ significantly (*p* > 0.05), whereas different uppercase letters indicate significant differences (*p* < 0.05). For paired comparisons within the same row (before vs. after fermentation), the absence of an asterisk (*) indicates no significant difference, while the presence of an asterisk (*) after fermentation indicates a significant difference (*p* < 0.05). A slash (/) indicates that no inhibition zone was detected.

**Table 3:** Size of inhibition zone of Chinese herbal to *S. flexneri* before and after fermentation.

Types of Fermented Herbs	Diameter of Inhibition Circle Before Fermentation (mm)	Diameter of Inhibition Circle After Fermentation (mm)	Types of Fermented Herbs	Diameter of Inhibition Circle Before Fermentation (mm)	Diameter of Inhibition Circle After Fermentation (mm)
Aspartame	/	/	*Herba japonica*	/	/
Dogwood	/	/	Gallnut	17.00 ± 0.00 ^B^	19.00 ± 0.00 ^A^*
Garlic	/	/	Wild chrysanthemum	7.50 ± 0.50 ^A^	7.50 ± 0.50 ^B^*
*Schisandra chinensis*	/	/	Ligustrum	/	/
Coptis	/	/	White atractylodes	/	/
Corydalis herb	/	/	*Radix isatidis*	/	/
Forsythia	/	/	Ginkgo leaf	/	/
*Polygonum cuspidatum*	/	/	Dandelion	/	/
Purslane	/	/	Schizonepeta	/	/
Catechu	7.00 ± 0.00 ^A^	7.00 ± 0.00 ^B^*	*Angelica sinensis*	/	/
Green bark	/	/	*Eucommia ulmoides*	/	/
Anemarrhena	/	/	Rhubarb	/	/
Chinensis	/	/	Red peony root	/	/
*Prunus chinensis*	/	/	*Eucommia ulmoides* leaf	/	/
Sapwood	/	/	hawthorn	/	/
Pulsatilla	/	/	Rehmannia	/	/
Myrobalan	16.50 ± 0.50 ^B^	14.50 ± 1.50 ^A^*	Licorice	/	7.50 ± 0.50 ^B^*
Wormwood leaf	/	7.00 ± 0.00 ^B^*	Sichuan dome	/	6.00 ± 0.00 ^B^*
Pomegranate peel	/	/	honeysuckle	/	/
Black plum	/	/	Green leaf	/	/
Scutellaria	/	8.50 ± 1.50 ^B^*	Cocklebur seed	/	/
Astragalus	/	/	andrographitis	/	/
Phellodendron	/	/	Soybean meal and starch	/	/

Note: Data are presented as mean ± SD. Within the same column, values sharing the same uppercase letter do not differ significantly (*p* > 0.05), whereas different uppercase letters indicate significant differences (*p* < 0.05). For paired comparisons within the same row (before vs. after fermentation), the absence of an asterisk (*) indicates no significant difference, while the presence of an asterisk (*) after fermentation indicates a significant difference (*p* < 0.05). A slash (/) indicates that no inhibition zone was detected.

**Table 4:** Feed efficiency of Chinese soft-shelled Turtle.

	Fermented Chinese Medicine Group (%)	Chinese Medicine Group (%)	Full Price Powder Group (%)
Feed efficiency	1.75 ± 0.03 ^b^	1.92 ± 0.07 ^a^	1.74 ± 0.03 ^b^
WG (g)	274.29 ± 3.6 ^a^	250.00 ± 4.3 ^b^	275.86 ± 3.4 ^a^
WG (%)	68.57 ± 0.72 ^a^	62.50 ± 0.85 ^b^	68.97 ± 0.69 ^a^
FCR	1.75 ± 0.02 ^a^	1.92 ± 0.03 ^b^	1.74 ± 0.02 ^a^
FI (g)	480.00 ± 0.4 ^a^	480.00 ± 0.4 ^a^	480.00 ± 0.4 ^a^
SR (%)	100	100	100

Note 1: Feed efficiency = feed consumption/weight gain × 100%. Note 2: Data are presented as mean ± SD. Within the same row, values sharing the same lowercase letter do not differ significantly (*p* > 0.05), whereas different lowercase letters indicate significant differences (*p* < 0.05).

**Table 5:** Comparison of RBC and WBC numbers of Chinese Soft-shelled turtle.

	Fermented Chinese Herbs Feed Group	The Control Group	Chinese Herbs Feed Group
WBC (10^9^/L)	10.21 ± 0.05 ^b^	28.73 ± 0.42 ^a^	9.76 ± 0.14 ^b^
RBC (10^12^/L)	3.64 ± 0.31 ^c^	2.62 ± 0.37 ^a^	3.09 ± 0.22 ^b^

Note: Data are presented as mean ± SD. Within the same row, values sharing the same lowercase letter do not differ significantly (*p* > 0.05), whereas different lowercase letters indicate significant differences (*p* < 0.05).

**Table 6:** Effect of compound fermented feed on biochemical indexes of Chinese soft-shelled turtle.

Items	Fermented Chinese Medicine Group (%)	Chinese Medicine Group (%)	Basal Diet Group (%)
ALT (U/L)	5.20 ± 0.15 ^b^	6.60 ± 0.33 ^b^	10.10 ± 0.24 ^a^
AST (U/L)	60.83 ± 0.44 ^c^	93.2 ± 0.45 ^b^	112.95 ± 3.44 ^a^
UREA (mmol/L)	0.92 ± 0.15 ^c^	1.23 ± 0.07 ^b^	2.62 ± 0.22 ^a^
UA (μmol/L)	44.00 ± 0.01 ^b^	50.40 ± 7.07 ^a^	50.50 ± 10.22 ^a^
CREA (μmol/L)	5.10 ± 0.03 ^b^	6.54 ± 0.07 ^a^	6.80 ± 0.12 ^a^
TG (mmol/L)	1.72 ± 0.02 ^b^	1.74 ± 0.15 ^b^	1.84 ± 0.11 ^a^
GLU (mmol/L)	5.22 ± 0.13 ^b^	5.70 ± 0.22 ^b^	4.24 ± 0.22 ^a^
TCHO (mmol/L)	8.55 ± 0.11 ^b^	5.24 ± 0.20 ^a^	5.60 ± 0.25 ^a^
HDL-C (mmol/L)	2.61 ± 0.14 ^b^	1.27 ± 0.07 ^a^	1.26 ± 0.06 ^a^
LDL-C (mmol/L)	5.28 ± 0.01 ^b^	3.68 ± 0.11 ^a^	3.53 ± 0.22 ^a^

Note: Data are presented as mean ± SD. Within the same row, values sharing the same lowercase letter do not differ significantly (*p* > 0.05), whereas different lowercase letters indicate significant differences (*p* < 0.05).

**Table 7:** Effect of compound fermented feed on serum antioxidant immune indexes of Chinese soft-shelled turtle.

Items	Fermented Chinese Medicine Group	Chinese Medicine Group	Full Price Feed Group
LSM (U/mL)	1033.22 ± 45.16 ^c^	817.32 ± 40.16 ^b^	533.1 ± 32.11 ^a^
T-AOC (U/mL)	3.54 ± 0.12 ^b^	3.29 ± 0.32 ^b^	2.30 ± 0.14 ^a^
ACP (U/mL)	1.84 ± 0.18 ^b^	1.84 ± 0.05 ^b^	1.88 ± 0.15 ^a^
AKP (U/mL)	4.49 ± 0.11 ^b^	4.33 ± 0.14 ^b^	5.55 ± 0.23 ^a^

Note: Data are presented as mean ± SD. Within the same row, values sharing the same lowercase letter do not differ significantly (*p* > 0.05), whereas different lowercase letters indicate significant differences (*p* < 0.05).

**Table 8:** Challenge experiment of *S. enteritidis*.

Group	Number of Experiment Turtles	Number of Surviving Turtles	Survival Rate
10% fermented Chinese medicine feed group	30	29	96.67% ^a^
10% unfermented Chinese medicine feed group	30	20	66.67% ^b^
Basal diet group	30	10	33.33% ^c^

Note: Data are presented as mean ± SD. Values sharing the same lowercase letter do not differ significantly (*p* > 0.05), whereas different lowercase letters indicate significant differences (*p* < 0.05).

**Table 9:** Challenge experiment of *E. coli*.

Group	Number of Experiment Turtles	Number of Surviving Turtles	Survival Rate
10% fermented Chinese medicine feed group	30	27	90.00% ^a^
10% unfermented Chinese medicine feed group	30	25	83.33% ^b^
Basal diet group	30	15	50.00% ^c^

Note: Data are presented as mean ± SD. Values sharing the same lowercase letter do not differ significantly (*p* > 0.05), whereas different lowercase letters indicate significant differences (*p* < 0.05).

**Table 10:** Challenge experiment of *S. fowleri*.

Group	Number of Experiment Turtles	Number of Surviving Turtles	Survival Rate
10% fermented Chinese medicine feed group	30	24	80.00% ^a^
10% unfermented Chinese medicine feed group	30	17	56.66% ^b^
Basal diet group	30	6	20.00% ^c^

Note: Data are presented as mean ± SD. Values sharing the same lowercase letter do not differ significantly (*p* > 0.05), whereas different lowercase letters indicate significant differences (*p* < 0.05).

**Table 11:** Challenge experiment of *Salmonella enteritidis*, *E. coli*, *Shilli fowleri*.

Group	Number of Experiment Turtles	Number of Surviving Turtles	Survival Rate
10% fermented Chinese medicine feed group	30	19	63.33% ^a^
10% Chinese medicine feed group	30	10	33.33% ^b^
Basal diet group	30	4	13.33% ^c^

Note: Data are presented as mean ± SD. Values sharing the same lowercase letter do not differ significantly (*p* > 0.05), whereas different lowercase letters indicate significant differences (*p* < 0.05).

## Data Availability

The available data set during and analyzed during the current study are available from the corresponding author upon reasonable request. The authors declare full data transparency.

## Supplementary Materials

The following supporting information can be downloaded at: https://www.mdpi.com/article/10.3390/ani16071054/s1, Table S1: Feed formulation for Chinese soft-shelled turtle.
