# Supplementary material for: Effects of Fermented Compound Chinese Herbal Feed on Gut Microbiota, Immune Response, and Disease Resistance in Chinese Soft-Shelled Turtle (Pelodiscus sinensis)
Source: Animals (Basel). 2026 Mar 31;16(7):1054. doi: 10.3390/ani16071054 (PMC13072027; doi:10.3390/ani16071054)
Supplement: Supplementary file 1 [file animals-16-01054-s001.zip › animals-4202186-supplementary.pdf]

SUPPLEMENTALLY TABLES

Table S1. Feed formulation for Chinese soft-shelled turtle.

Components	Fermented Chinese Medicine Group （%）	Chinese Medicine Group （%）	Full-price powder （%）
Full-price powder	87.7	87.7	97.7
Fermented compound herbal feed additives	10	0	0
Compound herbal feed additives	0	10	0
Multivitamins	0.2	0.2	0.2
Vc	0.1	0.1	0.1
Fish Oil	2	2	2

Note： Ingredients and Proximate Composition of the Full-price powder

1. Feed Ingredients (Full formulation list)

Fish meal, Soybean meal, Corn gluten meal,α-Starch, Wheat flour, Wheat gluten, Fish oil, Soybean lecithin, Dicalcium phosphate, Choline chloride, Vitamin premix (for soft-shelled turtle) , Mineral premix (for soft-shelled turtle) , Antioxidant, Pelleting binder.

The proximate chemical composition of the test diet was determined by standard analytical methods, and the analyzed values (expressed as mean ± standard deviation) are as follows: crude protein 46.0 ± 0.5%, crude lipid 8.5 ± 0.2%, crude ash 12.5 ± 0.3%, crude fiber ≤ 2.2%, moisture ≤ 10.5%, calcium 3.2 ± 0.1%, total phosphorus 1.8 ± 0.1%, and gross energy 19.6 ± 0.2 MJ/kg. All ingredients were purchased from qualified commercial suppliers and complied with the national feed safety standards (GB 13078-2017), ensuring the quality and safety of the experimental diet.
